# “Generality of mis-fit”? The real-life difficulty of matching scales in an interconnected world

**DOI:** 10.1007/s13280-015-0757-2

**Published:** 2016-03-03

**Authors:** E. Carina H. Keskitalo, Tim Horstkotte, Sonja Kivinen, Bruce Forbes, Jukka Käyhkö

**Affiliations:** 1 Department of Geography and Economic History, Umeå University, 901 87 Umeå, Sweden; 2 Department of Geography, University of Turku, 20014 Turku, Finland; 3 Arctic Centre, University of Lapland, 96101 Rovaniemi, Finland

**Keywords:** Goodness of fit, Scale, Mismatch, Reindeer husbandry, Fennoscandia

## Abstract

A clear understanding of processes at multiple scales and levels is of special significance when conceiving strategies for human–environment interactions. However, understanding and application of the scale concept often differ between administrative-political and ecological disciplines. These mirror major differences in potential solutions whether and how scales can, at all, be made congruent. As a result, opportunities of seeking “goodness-of-fit” between different concepts of governance should perhaps be reconsidered in the light of a potential “generality of mis-fit.” This article reviews the interdisciplinary considerations inherent in the concept of scale in its ecological, as well as administrative-political, significance and argues that issues of how to manage “mis-fit” should be awarded more emphasis in social-ecological research and management practices. These considerations are exemplified by the case of reindeer husbandry in Fennoscandia. Whilst an indigenous small-scale practice, reindeer husbandry involves multi-level ecological and administrative-political complexities—complexities that we argue may arise in any multi-level system.

**Electronic supplementary material:**

The online version of this article (doi:10.1007/s13280-015-0757-2) contains supplementary material, which is available to authorized users.

## Introduction

A key challenge in environmental management arises from the need to simultaneously consider and understand the variability of, and interactions within, biophysical systems and those that originate from social institutions—often jointly referred to as social-ecological systems (SES, Berkes and Folke 1998). SESs are characterised by coevolution between and within their two elements: change in the one results in change in the other, continuously causing further change. These mutual influences are referred to as complexity (Holling 2001). Social-ecological systems integrate many different *scales*, i.e. explanatory dimensions for observed processes. These include spatial (the archetype scale), temporal, jurisdictional, institutional or knowledge scales, with numerous feedbacks and interactions across these (Cash et al. 2006). Each scale (e.g. spatial) is composed of hierarchically organised measurement units, termed *levels* (e.g. global, regional, local) (Gibson et al. 2000; Cash et al. 2006). Scale and level are, therefore, important concepts in approaching SESs i.e. considering social elements (“people”) and ecological elements (“nature”) as one coherent system.

In order to bring these components into accord, it is often argued that a “goodness-of-fit” between governance in its ecological and social components and dynamics must be sought (Folke et al. 2007). “Fit” has gained increased attention as the general aim in the management of human-environment interactions, though the focus has been on rather specific resource regimes such as water governance (e.g. Young 2002). However, it has also been suggested that all relevant institutions in relation to any given ecosystem should be included (e.g. Ekstrom and Young 2009). Thus, to achieve a successful goodness-of-fit, for instance Cumming et al. (2013) emphasise the necessity of institutions being flexible and “at appropriate scales to strengthen feedbacks that modify and moderate demand for ecosystem services and incorporate the trade-offs between human wellbeing, profit, and the exploitation of ecosystems” (see also Forbes et al. 2009). Despite this aim, processes towards such goodness-of-fit typically stall in their infancy (Hein et al. 2006): mis-matches often occur concerning how scales and levels are conceived and used as analytical tools in social-administrative disciplines on the one hand, and in ecology on the other.

The focus of SES work has often been on lower organisational levels (Ernstson et al. 2010), which may be regarded as more easily manageable and less complex than higher organisational levels. However, archetypal local livelihoods such as hunting, fishing and other renewable, resource-based practices are also regulated by institutions at higher hierarchical levels, for example by the European Union. Multiple-use situations of natural resources, where a landscape and/or its resources are used by different stakeholders, involve additional challenges due to differing management goals and strategies (Forbes et al. 2006; Hein et al. 2006). Here, development of potentially satisfactory fit of governance with ecological patterns and processes for one land user might impact negatively other land users with different resource management scopes. As a consequence, it may not be possible to manage local conflicts by considering the local level only, nor by assuming that organisation will relate itself to ecological processes or be able to fit with these (e.g. O’Brien and Leichenko 2000; Næss et al. 2005).

Following the argumentation above, we contest the standard assumption that ‘fit’ is necessarily possible in complex systems, including at the local level. We base this on the complexities (sensu Holling 2001) of the multiple scales and levels that influence land uses, and that might prevent a coherent understanding of what is required for the governance of SESs. Instead, we suggest that a “generality of mis-fit” is the rule rather than the exception in the governance of SES. The dilemma of “managing mis-fit” could be a more realistic—but no less demanding—governance challenge that must be successfully addressed, rather than efforts to create idealistic outcomes.

We first review how underlying concepts and challenges to understanding of scales and levels as crucial components of any SES diverge from each other in ecology and administrative-political theory, i.e. the potential of mis-fit or mis-matches within these disciplines mirrored in formal decision-making. We then illustrate the challenges of mis-fit in managing SESs using the case of indigenous reindeer husbandry in northern Fennoscandia, where we discuss the barriers to conformity between scales and levels from various ecological and administrative-political aspects, as well as from the views of the various actors and institutions involved (including e.g. forestry and state level regulation). In particular, we illustrate (i) spatial mis-matches where a phenomenon at one level, e.g. regional or local, does not fit with it at another, (ii) temporal mis-matches between levels, e.g. slow and fast dynamics, and (iii) functional mis-matches, where the scope of solving a problem does not fit with the process causing the problem (Cumming et al. 2006; Guerrero et al. 2013). We conclude that these types of complexities will most probably be common to any complex, multi-level system and that more emphasis on how to govern the “generality of mis-fit” both in research and in management practices is necessary.

## The conceptualisation of scale in ecological systems

As ecological processes can be described at a variety of spatial and temporal hierarchical levels, there is no self-evident “natural” set of scales and inherent levels at which ecological phenomena should be studied (Levin 1992; Wiens 1989). When analysing ecological processes, two components are commonly considered: the grain and the extent of the phenomenon studied. Grain refers to the size of the individual units of observation that compose the sampling unit, while the extent of the phenomenon refers to the geographical space and/or duration of time over which comparisons are made (Turner et al. 2001; Rahbek 2005).

Perhaps because natural world phenomena are observable and measurable, ecological processes have often been assumed to be primary to SES, and to be the processes to which other processes should refer (e.g. Silver 2008). However, the degree of variability in ecological systems is in itself considerable and includes numerous potential mis-matches. As grain and extent set the lower and upper limits of spatial and temporal resolution when describing patterns and processes, the magnitude and direction of a given process and the patterns it creates may change with the alteration of both grain and extent (Wu 2004). Consequently, any observed process and resulting pattern depends on the particular scale and level of observation (Rahbek 2005). If the grain of analysis is enlarged while keeping the extent constant, the variance of a process will decrease because processes at lower levels will be averaged out at coarser resolution (Wiens 1989; Levin 1992). Furthermore, enlarging the spatial or temporal extent of a given process will increase its heterogeneity in space and/or time (Wiens 1989). For example, increasing the spatial extent of a study area may help resolving the ranges of species and the factors controlling hierarchically higher distribution patterns, whereas a more limited extent may help understand local population patterns and their determinants. Ecological amplitudes (sensu Ellenberg 1988) of individual plant and animal species may vary widely, with the result that sampling unit size ultimately depends on the aims of the study. This complicates the comparison of ecological studies carried out using different grains and extents of study. Furthermore, extrapolations of the results for predicting phenomena at other spatial and/or temporal dimensions are complex and risky (Miller et al. 2004), and may result in potential spatial mis-matches.

In addition, both grain and extent are often chosen subjectively, which means that two careful, well-meaning scholars can disagree over the appropriate approach to selecting spatial and temporal levels, as well as the choice of the sampling interval in time and space. For example, within a chosen grain, the respective environmental gradients across which the constituent species within a given community or ‘association’ occur must also be dealt with. Gradients between different communities or ecosystems (called ecotones) may be abrupt or gradual, depending on e.g. the changing competitive advantages of one community over the other or on abiotic factors such as elevation gradients (Cadenasso et al. 2003). Shifting environmental conditions change competitive advantages between neighbouring communities and introduce a dynamic pattern in their extent and pattern. However, the concept of ecotones is also inherently determined by human attitudes and interpretation of ecological phenomena (Whittaker 1956). These possible variations in how to choose grain and extent necessarily means that there is also a risk of functional mis-matches, i.e. that studying a phenomenon at a lower level will ignore those at higher levels.

Finally, in the ecological system, slow temporal processes at higher hierarchical levels constrain lower-level, faster processes (Allen and Hoekstra 1990). According to Pielou (1988), slower processes taking place over large spatial dimensions comprise the domain of biogeography, whereas ecology treats faster processes at lower spatiotemporal hierarchical levels. However, the distinction between these two disciplines is often blurred. The shift from one level to another is not linear, as spatiotemporal patterns observed at each level are controlled by different biotic and abiotic processes. For example, individual plant processes are related to site-specific conditions and local patch dynamics regulated by plant competition, observed at low spatiotemporal levels. Ecosystem dynamics are controlled by processes acting at higher spatiotemporal levels, such as fire or forestry. Abiotic conditions, such as climate or geological properties form the basis of ecosystem structure and functioning and are driven by processes extending to even larger spatial and longer temporal extents. As a result of these spatiotemporal considerations in ecological research, the earlier paradigm of ‘ecological equilibrium’ has been replaced by the ‘dynamics of nature’ (Wu and Loucks 1995). This also results in the possibility of functional mis-matches within the ecological system, and that all three categories of mis-matches are possible in the ecological system alone.

## Concepts of scale in administrative-political systems

SES research has often argued that social systems should be organised in relation to ecological systems (e.g. Silver 2008). To gain “fit” here relates to the fact that units in the administrative-political^1^ system are commonly not based on ecological organisation only, but on political, economic and other interests and interactions that have occurred over millennia. Administrative-political systems constitute a different logic to that of ecological systems, and developed historically from city states and feudal kingdoms into today’s multi-level governance systems. Multi-level governance is defined as the participation of different actors at different levels in decision-making and aims to serve as an, often descriptive, term for this complexity, including sub-national, national and supranational, as well as private and non-governmental interests (Marks and Hooghe 2004).^2^ In comparison with a focus on grain and extent in ecological scale, thus, the pattern of governance or steering in these systems differs widely in local, regional, national and international configurations between cases in or related to different countries, as numerous different regimes on different levels—such as international trade, the general broader institutions of the state, or specific regional or local configurations—influence any given resource use. As no single, generally acceptable conceptualisation of units can thereby be found, descriptions of the administrative-political system regularly utilise different theoretical conceptions to study specific cases, and to define what is regarded as important in these. This variation in study cases can be seen as one reason for the extensive variation in terms of formal theories that exist in social sciences. Consequently, SES research has to become more scale-sensitive and regard scale not only as falling under “objective and universal laws” (Padt and Arts 2014, p. 9).

As a result, the socio-economic and political system must be seen as one where scale, i.e. the explanatory dimension, is not a given but as one where scale is constructed differently by different actors, with different features highlighted in various theoretical conceptions (see e.g. Bulkeley 2005). Spatial, temporal and functional mis-matches are thus common: due to the complexity of the socio-economic and political system, any phenomenon related even to specific cases or areas for natural resource management will probably have somewhat different definitions at different levels (e.g. at the European Union level compared with specific local level). Phenomena may also have different temporal dynamics where, for instance, developing new legislation at national level or changing local practices is often slow, but may be hastened by other impacting factors (e.g. intersecting processes of developing legislation in other sectors). As a result of these processes and the different conceptualisations of problems at different levels, the very problem that is to be solved may thus be defined differently at higher versus local levels among the different actors involved, causing a functional mis-match. For instance, matching management goals may be constrained by administrative boundaries and thus call for joint, cross-border management. Such boundaries that can affect resource management and policies in economic and legal manners range from local level, such as land ownership (Nonaka and Spies 2005), to regional level, national borders and beyond (Forbes et al. 2006; Plagányi et al. 2013). The magnitude of these functional mis-matches may vary with the frequency, severity, extent and duration of events (for instance, policy, political, natural disaster or other, cf. Kingdon 1995) that influence the prevalent dynamics of the political-administrative system in general. Also, power relationships among different interest groups and their institutions often result in the maintenance of specific resource access rights, making resource access an inherently political issue. In order to manage this situation in their own interests, actors may for instance “jump scale” in an attempt to influence their local resource regime through participation in international processes that may have an impact locally (Gupta 2008). The equity in terms of the result of such a complex construction of scale depends on numerous factors, including which consequences for which different stakeholders are recognised or ignored (Adger et al. 2005).

By definition, thus, multi-level governance systems in comparison with more state-based governance often lack one clear absolute authority or clear hierarchy (Hooghe and Marks 2001; Piattoni 2010), allowing both for a devolution of authority e.g. upwards from the state to the EU, and downwards from the state to local levels. In one of the various traditions existing in the political science field, authors have defined such complex decision-making systems as polycentric, with the definition that units in such systems may be able to make mutual adjustments rather than only rely on, for example, increased governmental control or decentralisation (e.g. Nagendra and Ostrom 2012). However, the assumption on mutual adjustment or learning through polycentricity has commonly been criticised for ignoring the risks of free-riding, limited accountability and information asymmetry, as well as failing to recognise the risk that public officials and interests groups may act more in their own interest than in the interests of readjusting the system as a whole (e.g. Araral and Hartley 2013). It thus needs to be recognised that polycentric, multi-level governance systems may also create structures where economically more powerful interests may more easily gain a stronger role, while interests lacking time and funding for participation may lose out (see e.g. Pateman 1970; Nanz and Steffek 2004; Jentoft 2006). Given that complex governance is impacted not only by issue-specific aims, the existence of multiple aims within the systems will also inherently limit possibilities for alignment.

As a result, the development and application of an understanding of the scale concept in social systems (including broad administrative-political systems) necessitates embracing the complexity inherent in these systems, rather than assuming that re-ordering of processes will take place autonomously (cf. Nagendra and Ostrom 2012; Araral and Hartley 2013).

## Case study: Addressing “mis-fit” of scale and level in reindeer husbandry

The ecological understanding of scale as a context-specific combination of grain and extent in time and/or space is difficult to translate into the fluid, evolving and actor-dependent conceptualization of scale identified above in socio-administrative systems. The resulting complexities in re-organising natural resource management to fit ecological and political-administrative processes are apparent in the management of multiple-use natural resources, with its common conflicts of interest between different stakeholders.

**Fig. 1:**
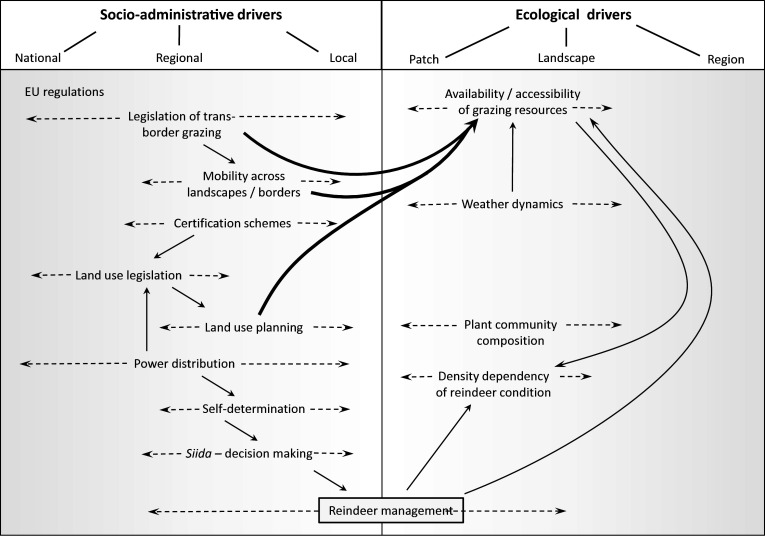
Socio-administrative and ecological processes that directly or indirectly affect decision-making in reindeer husbandry. Drivers vary in their importance across hierarchical levels (dashed arrows) and may influence each other (*solid arrows*). Several drivers of socio-administrative origin impact ecological drivers (*bold arrows*). See 10.1007/s13280-015-0757-2 for further details

As illustration, we focus on mis-fits of scale in decision-making in Saami reindeer husbandry in Fennoscandia that originate from ecological and administrative-political drivers. Especially, we analyse how reindeer husbandry, by interaction with other land users, is affected in its land use, productivity and economy, as well as in its governance and problem-solving capacity. Figure 1 illustrates the diversity and interaction of significant ecological and socio-political processes and factors that influence decision-making in reindeer husbandry. In particular, Fig. 1 emphasises the different levels across which these factors act (see 10.1007/s13280-015-0757-2 for details). We chose to examine reindeer husbandry as this form of livelihood includes local and traditional indigenous scales of decision-making, often highlighted in SES conceptions (e.g. Ernstson et al. 2010). However, reindeer husbandry today is a complex land use system where the primary income comprises the sale of reindeer meat, often to suppliers in Norway, Sweden and Finland. As a semi-domestic animal, reindeer graze freely for the main part of the year, requiring a diverse selection of seasonal grazing grounds. The herding area covers large parts of the countries where it is practised—for example, about 40 % of the land area and several counties in Sweden—including areas with major infrastructure development and urban areas of different sizes. This space requirement brings about conflict with almost every other land use due to their impact either on the reindeer migration routes or on grazing areas.

### Land use aims and strategies among the various actors, as manifested in the landscape

**Fig. 2:**
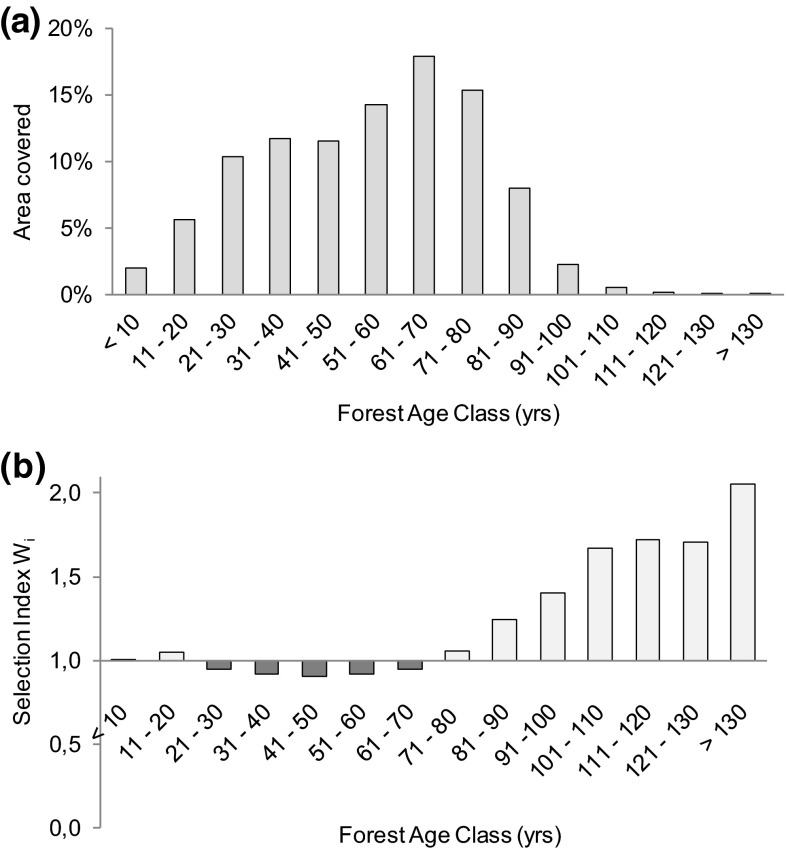
Characteristics of Norrbotten’s boreal forest in the reindeer winter grazing area. **a** Area coverage of forest age classes. **b** Selection index *W*_*i*_ for forest age classes, indicating forest age classes that are preferred by reindeer. The index is derived by the equation: *W*_*i*_ = [(% area covered by forest age class *i* in the areas preferred by reindeer)/(% area covered by forest age class *i* in the entire study area)]. Values larger than 1 indicate preference (*light bars*), values less than 1 indicate avoidance (*grey bars*). See 10.1007/s13280-015-0757-2 for further details

As early as the descriptions of the physical area, variations exist between how different land uses and systems define use, i.e. a functional mis-match. In general, reindeer husbandry is affected by the dynamics of landscape composition in multi-use environments that affect seasonal availability and accessibility of foraging resources, such as lichen-rich old growth forests (Kivinen et al. 2010). The spatial and temporal composition of the landscape, i.e. the changing abundance of different forest age classes with specific structures and functions for reindeer grazing (Roturier and Roué 2009), is strongly regulated by institutional design, such as land ownership and rotation of forest harvest. In northern Sweden, the majority of forest age classes currently range between 70 and 90 years (with final logging commonly undertaken after about 70–120 growth years). As a result, in the winter grazing areas in e.g. Norrbotten (the northernmost county of Sweden) forestry practices have led to a heavily fragmented landscape with low supply of old growth forests older than 120 years, and targeted towards forestry-related production characteristics. Today, 73 % of forest stands in this county are younger than 70 years (Fig. 2a, see 10.1007/s13280-015-0757-2 for details).

**Fig. 3:**
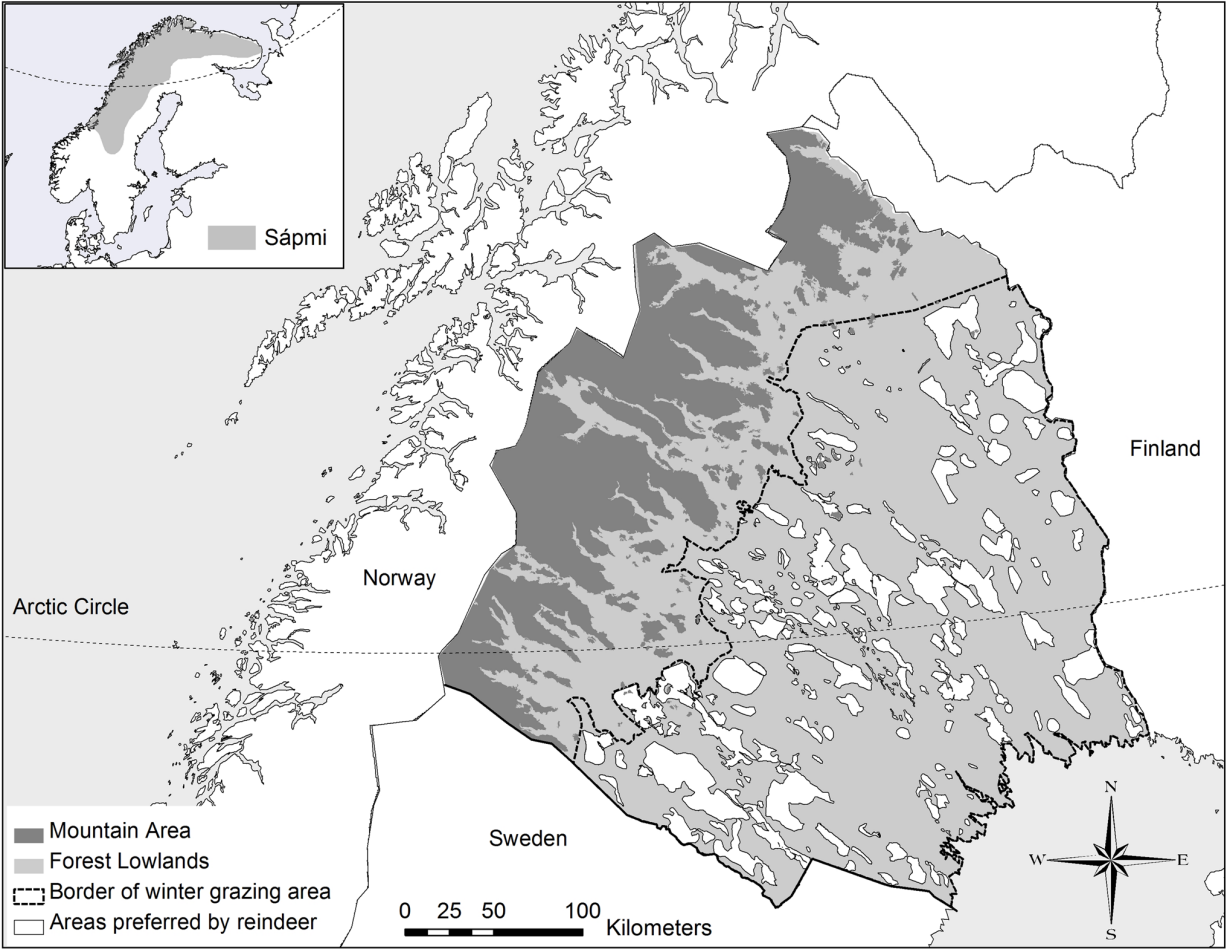
Map of Norrbotten. The dotted line represents the approximate border of the winter grazing area in the forest lowlands. Areas preferred by reindeer (“trivselland”) are shown by *white areas*. The *inset* map in the *upper left* illustrates Sápmi as the ancestral area of Saami peoples

Relative to the availability of forests stands older than 80 years, areas preferred by reindeer contain disproportionately many of these stands. This ratio (selection index *W*_*i*_, Manly et al. 2002, see 10.1007/s13280-015-0757-2 for details) indicates that reindeer prefer older forests (Fig. 2b). In Norrbotten, individual forest stands cover a median area of 5 ha. In comparison, the median of areas reflecting behavioural preferences by reindeer, e.g. for grazing, calving or resting (“trivselland”; Norrbotten County Administrative Board) covers 2197 ha (Fig. 3). Strong contrasts therefore exist in landscape patterns created by forestry compared to the needs of reindeer grazing and migration, exemplifying spatial and temporal mis-matches in landscape management based on varying management aims among different actors (Horstkotte et al. 2014).

### Inability to reach agreement on ecological limits or to develop purely local management

In relation to these varying land use strategies, the risk of forage depletion by overgrazing due to larger than permitted herds, whose size is based rather on traditional values of reindeer husbandry has been emphasised, but also contested in northern Norway, Sweden and Finland alike. Also climatic conditions and vegetation interaction may complicate the relationship between reindeer herd size, animal weight, grazing pressure and vegetation recovery (Tømmervik et al. 2012). The situation is complicated by the fact that limited recovery of grazing resources may also be attributed to other forms of land use, such as tourism or forestry, especially in the reindeer winter grazing grounds in Finland and Sweden. This situation can be regarded as a result of a functional mis-match as it is difficult to assess the effects different land uses may have. In trying to manage the situation, different strategies, such as supplementary feeding, have been instituted to maintain carrying capacity by compensating for the loss of arboreal lichens resulting from extensive forestry (Helle and Jaakola 2008). Yet, even as such steps have been implemented for decades, carrying capacity and ‘overgrazing’ remain elusive concepts (Mysterud 2006; Forbes and Kumpula 2009). Depending on the philosophy of determining carrying capacity, the time for decision-making is crucial. From the authorities’ perspective, carrying capacity may represent a fixed relationship between available forage resources and reindeer production, while herders perceive carrying capacity as extremely dynamic due to e.g. environmental variation and herd size of neighbouring *siida* (Reinert and Benjaminsen 2015). Clearly, the concept of carrying capacity is value-laden (cf. Mysterud 2006), and cuts across ecological, political, economic and cultural conceptions. Any solution for the dilemma must therefore be prepared to address the different spatial, temporal and functional problem definitions and different scales that result in different mis-matches (Rees et al. 2008).

The possibilities of resolving such varying conceptions, e.g. carrying capacity, through management at local level is limited, since actors competing with reindeer husbandry and making decisions on local land use are, to a large extent, non-local. Today, reindeer husbandry is practised by a relatively small number of herders representing a minority of the Saami population; in Sweden by some 2000–2500 individuals. On a national level, compared with other land uses in these areas, the livelihood thus provides negligible economic income: forestry, in northern Sweden practised largely on the same grounds, provides some 3 % of GDP or 10 % of national Swedish export value also with some benefit to the small-scale, individual, forest owners who own a large proportion of Swedish forest (Keskitalo 2008b; Keskitalo et al. 2014). Potentially as a result, very limited changes in power distribution between the different land uses in the case of forestry and reindeer husbandry have taken place over time (Keskitalo 2008a). However, income generated by reindeer husbandry can be of great importance at local levels and is, for example, of considerable cultural importance. There are thus very strong economic differences between sectors, and as one of the smallest sectors economically and by numbers of participants, reindeer husbandry is strongly affected by all other land uses over these larger areas. Forestry, for instance, includes the full variety from local forest owners to national and supranational companies, while mining is predominantly run by national and supranational companies. In the case of supranational companies, decision-making is regularly removed from the local level, and local consultation discussions between, for example, forestry and reindeer husbandry are thus regularly governed by higher-level company aims (Keskitalo 2008b). A high potential for mis-matches across levels and scales therefore exists when designing strategies for sustainable multiple-use management of landscapes and resources that are used by reindeer husbandry and other forms of land use simultaneously, and providing benefits for different local groups as well as individuals. However, it should also be noted that whilst the relationship between sectors may be conflictual at higher hierarchical levels many local groups, including reindeer herders, may be active in several of the sectors and maintain non-conflictual relationships on the individual level: local people in Sweden, including non-Saami, may own reindeer that are herded by Saami (*skötesrenar*), while reindeer herders may be employed e.g. in forestry or mining (e.g. Keskitalo 2008a, b).

### The legal multi-level system as a battleground for differing conceptions

Given these spatial–temporal mismatches at local and regional levels, the state as a body determining and enforcing national law must manage and include multiple different land use conceptualisations within its regulative framework. Today, the state organisation at several administrative-political levels considers reindeer husbandry as one land use amongst other land uses. Administrative levels range from national level management to regional and local implementation. Local implementation focuses on specific districts (in Norway and Finland) or reindeer husbandry administrative units (in Sweden). Based on ecological and socio-economic requirements, the number of reindeer is limited to set boundaries (related to carrying capacity assessments amongst other aspects) and assumes a relatively equalised distribution of reindeer between the reindeer husbandry administrative units. However, in all cases, these districts or units differ from the smaller, family or kinship-based units of traditional organisation in reindeer husbandry, in the traditional Saami *siida* system. The *siida* system reflects family ties and historical reindeer herd size in which considerable discrepancies in herd sizes may exist between families. In that it is very small-scale, it has historically provided for flexibility in moving the herds depending e.g. on grazing conditions (Brännlund 2015). Today, all districts/reindeer husbandry administrative units include several *siida*; however, the formal authority of the *siida* systems varies between countries: *siida* systems are recognised as legal units in Norway but are informal elements of the broader reindeer husbandry unit in Sweden, which is the unit to which land areas are allocated for use. In the current situation of land use change, the *siida* thus no longer provides the same flexibility in reindeer husbandry as compared to earlier strategies, and the formal system results in unclear *siida* rights in land use decisions and property rights (Sara 2011). This situation thus creates spatial (specifically administrative and legal) mis-matches with definitions of reindeer herding units varying between levels.

As a result of these complexities—potentially also exacerbated by the fact that climate change may further worsen the situation for reindeer husbandry (e.g. Moen 2008)—multiple attempts at “jumping scale” (cf. Gupta 2008) to gain support for reindeer husbandry at higher levels have taken place. Thus, with regard to reindeer husbandry for example decisions at the level of the ILO Convention No. 169—the international convention on indigenous peoples’ rights to land—as well as decisions in the UN Indigenous peoples’ forum have been utilised to impose pressure on states. Contrastingly, states highlight their national sovereignty and complex local dynamics where they need to consider multiple interests. Such considerations have resulted both in discussions over the protection of indigenous peoples’ rights, for example the right to self-determination and ownership of land, in accordance with international legislation and in discussions on how states are to manage these multiple pressures locally (e.g. Keskitalo 2008b). Such dynamics illustrate that functional mis-matches are often taken to different authorities in attempts to define an authoritative manner of managing the problem.

## Discussion and conclusion

This study has illustrated the challenges in developing and combining conceptions of scales originating from ecology with conceptions of scales originating from administrative-political disciplines. Indeed, both types of conceptions of scales also include internal mis-matches. For example, the ecological system does not have one obvious combination of grain and extent, holds large variation (e.g. large habitat variation within a biome) and it is difficult to extrapolate phenomena to other levels than that observed (e.g. long-range dissemination of locally observed species). Social or administrative-political conceptions of scales vary by case and necessitate consideration of the construction of scale rather than treating it as given.

Our case study of cross-scale management at a multitude of levels relevant to reindeer husbandry in Fennoscandia identified all three types of mis-matches (Cumming et al. 2006; Guerrero et al. 2013). With regard to spatial mis-matches, local definitions of herding units in the traditional *siida* system do not fit with either the ecological definitions, or with higher-level administrative division of the reindeer husbandry area by the relevant states (cf. Brännlund 2015). Temporal mis-matches in the multiple-use situation of boreal forests are evident by the rotation times practised in forestry causing old-growth forests, which are important habitat types for reindeer, to disappear. At the same time, the administrative-political system operates in jurisdictions with sometimes limited connections between them, and they are commonly not organised in relation to ecological processes. Taken together, these processes illustrate functional mis-matches where the scaling of administrative-political systems and selection of actors vary widely depending on the specific case and the power structure among the actors. As a consequence, problem descriptions at local and higher levels fail to relate to each other. Functional mis-matches between different scales become particularly evident when these scales represent different views on what principles should govern management decisions. In fact, our analysis of reindeer husbandry illustrates factors that lie beyond regular conceptions of functional mis-matches (e.g. Dallimer and Strange 2015): the deviation of problem definitions, or so called framing, amongst for example various different authorities and interests that enforce how specific conflicts are addressed (e.g. Bulkeley 2005).

Thus, given the nature of the multi-level governance system, these potential solutions often identify various authorities, thereby developing specific, often competing, solutions: managing rather than “solving” problems in any more definitive sense. These dynamics thereby go beyond a more limited definition of functional mis-matches as purely one where the scope of solving a problem does not fit with the process causing the problem. Rather, conflicts are results of disagreements over the framing of the problem and subsequently which decision-making and rights principles should apply. This makes mis-matches not only an epistemological barrier, but also an institutional or systemic barrier. Often conclusions on mis-fit of scales and the resulting consequences derive from relatively self-organising systems at lower hierarchical levels (cf. Nagendra and Ostrom 2012). However, under the current extent of globalisation, we argue that almost no systems remain apart from high level hierarchical and more general (national and international, including both governmental/public and private industry/corporate) organisation. As a consequence, multiple organisational levels will exist in each given case, and will largely express understandings and priorities other than those of local or even regional levels. These various arrangements, even related to one single resource, sometimes do not align. Whether reasons for this may be related to interest, power, or variations in terms of aims in other senses, autonomous realignment (cf. Nagendra and Ostrom 2012; Araral and Hartley 2013) cannot be assumed.

Thus, we emphasise that in particular where resource management includes not only the governance of one single resource, but rather multi-use and multi-interest situations, “fit” between scales will more often be a question of political compromise between parties involved, rather than of a one single good solution. In such cases, management of mis-match—including managing and clarifying different conceptions of a problem—may often be a more realistic point of departure than assuming that goodness of fit can be identified. These kinds of dynamics should then be expected in numerous cases of natural resource management, and are in fact highlighted in much social science literature on problem framing and agenda setting (see e.g. Schön and Rein 1994; Kingdon 1995 for some seminal works). Ecological and SES literature in particular suggest that management should be compatible with critical ecosystem functions (Walters 1986; Holling 1996). Yet the possibility to truly make scales congruent, to cover all scales that are appropriate, or to achieve goodness of fit between them may be more the exception than rule—or at the least not be possible to assume.

As a result, the reality that decision-making between different sectors, as well as at high hierarchical levels may not be able to (or willing) to re-orient towards lower levels or specific cases may have been allocated too little focus in SES research and related management. Understanding varying framings, political and economic power and inherent complexity—not assumed as automatically organising towards similar aims (cf. Araral and Hartley 2013; Padt et al. 2014)—must thus be a crucial focus in developing methods to work with mis-matched systems. Established social science understandings of the role of more general governance regimes, the influence of governance systems not centred upon the particular resource in focus for any study or management, and the fact that all interests may not wish to learn or align in relation to certain specific aims thus have to be included in SES analysis in order to understand the actual problems of fit (see e.g. Wellstead et al. 2013).

## Electronic supplementary material

Below is the link to the electronic supplementary material.

Supplementary material 1 (PDF 461 kb)

## Footnotes

1 The term "administrative-political" is chosen here to reflect the focus in this section on political science-related literature: given the complexity within social and human sciences a description centred, for instance, on anthropological concepts would highlight different conceptions. The focus on a system description related to political science concepts has here been chosen to reflect the formal decision-making properties of systems at different levels (ranging from local to international) that impact decisions on local resource use. If anything, the description here thus underplays rather than overplays complexity, as many additional conceptions of social systems could be included.

2 Here, for example, re-organisation of the political system and its shifting as well as fluid scale is defined through “Type I” governance, i.e. historical organisation into rather fixed local, regional, national and international units, in comparison to “Type II” governance, seen as more prevalent today (Hooghe and Marks 2001; Marks and Hooghe 2004). Compared with a more traditional division focused on the state, Type II includes private actors and is characterised by a patchwork of many, partially overlapping jurisdictions that may even include “dispersed self-rule on the part of diverse voluntary groups” (Marks and Hooghe 2004).
