# Supplementary material for: “Generality of mis-fit”? The real-life difficulty of matching scales in an interconnected world
Source: Ambio. 2016 Mar 3;45(6):742–52. doi: 10.1007/s13280-015-0757-2 (PMC5012993; doi:10.1007/s13280-015-0757-2)
Supplement: Supplementary file 1 — Supplementary material 1 (PDF 461 kb) [file 13280_2015_757_MOESM1_ESM.pdf]

Ambio

Electronic Supplementary Material

This supplementary material has not been peer reviewed.

Title: "Generality of mis-fit"? The real-life difficulty of matching scales in an interconnected world

Authors: E. Carina H. Keskitalo, Tim Horstkotte, Sonja Kivinen, Bruce Forbes, Jukka Käyhkö

S1: Table S1 - Socio-administrative and ecological drivers influencing decision making in reindeer husbandry

Figure 1 in the main text illustrates the main drivers of ecological and socio-administrative origin that affect reindeer husbandry across several levels. These have been identified from the literature, often referred to also later in our manuscript. Below, we specify which references specifically address the identified topic and helped us to develop Fig. 1.

Table S1: Main drivers affecting reindeer husbandry across several levels

**Socio-administrative drivers**	**References**
EU regulations	Johansson 2008, Keskitalo 2008a
Legislation of trans-border grazing	Brännlund & Axelsson 2011, Lantto 2010
Mobility across landscapes / borders	Lantto 2010, Brännlund & Axelsson 2011, Horstkotte *et al.* 2014
Certification schemes	Keskitalo 2008b, Johansson 2014
Land use legislation	Sandström *et al.* 2003, Widmark 2009
Land use planning	Sandström *et al.* 2003, Herrmann *et al.* 2014
Power distribution	Sandström *et al.* 2006, Ulvevadet 2008, Löf 2014
Self-determination	Axelsson & Sköld 2006, Allard 2014
Siida decision making	Sara 2009, 2011
Reindeer management	Tyler *et al.* 2007, Forbes *et al.* 2006

**Ecological drivers**	**References**
Availability / accessibility of grazing resources	Helle 1984, Kivinen *et al.* 2010, Skarin *et al.* 2010
Weather dynamics	Helle & Kojola 2008, Kumpula & Colpaert 2009
Plant community composition	Eskelinen & Oksanen 2006, Bråthen *et al.* 2007
Density dependency of reindeer condition	Kumpula *et al.* 2000, Tveraa *et al.* 2007, Bårdsen & Tveraa 2012

References:

Allard, C. 2014: The Nordic countries' law on Sámi territorial rights. *Arctic Review, 2*(2).

Axelsson P. & Sköld P., 2006: Indigenous Populations and Vulnerability. Characterizing Vulnerability in a Sami Context, *Annales de démographie historique*, 115-132.

Bråthen, K. A., Ims, R. A., Yoccoz, N. G., Fauchald, P., Tveraa, T., & Hausner, V. H. 2007: Induced shift in ecosystem productivity? Extensive scale effects of abundant large herbivores. *Ecosystems, 10*(5), 773-789.

Brännlund, I., & Axelsson, P. 2011: Reindeer management during the colonization of Sami lands: A long-term perspective of vulnerability and adaptation strategies. *Global Environmental Change, 21*(3), 1095-1105.

Bårdsen, B. J., & Tveraa, T. 2012: Density-dependence vs. density-independence–linking reproductive allocation to population abundance and vegetation greenness. *Journal of Animal Ecology, 81*(2), 364-376.

Eskelinen, A., & Oksanen, J. 2006: Changes in the abundance, composition and species richness of mountain vegetation in relation to summer grazing by reindeer. *Journal of Vegetation Science, 17*(2), 245-254.

Forbes, B. C., Bölter, M., Müller-Wille, L., Hukkinen, J., Müller, F., Gunslay, N., & Konstantinov, Y. (Eds.). 2006: *Reindeer management in northernmost Europe: linking practical and scientific knowledge in social-ecological systems* (Vol. 184). Springer.

Helle, T. 1984: Foraging behaviour of the semi-domestic reindeer (Rangifer tarandus L.) in relation to snow in Finnish Lapland. *Rep. Kevo Subarct. Res. Stn.*, *19*, 35-47.

Herrmann, T. M., Sandström, P., Granqvist, K., D'Astous, N., Vannar, J., Asselin, H., *et al*. 2014: Effects of mining on reindeer/caribou populations and indigenous livelihoods: community-based monitoring by Sami reindeer herders in Sweden and First Nations in Canada. *The Polar Journal*, *4*(1), 28-51.

Horstkotte, T., Sandström, C., & Moen, J. 2014: Exploring the Multiple Use of Boreal Landscapes in Northern Sweden: The Importance of Social-Ecological Diversity for Mobility and Flexibility. *Human Ecology*, *42*(5), 671-682.

Johansson, J. 2014: Towards democratic and effective forest governance? The discursive legitimation of forest certification in northern Sweden. *Local Environment*, *19*(7), 803-819.

Johansson, P. 2008: *Samerna-ett ursprungsfolk eller en minoritet? En studie av svensk samepolitik 1986-2005*.PhD thesis. Gothenburg.

Keskitalo, E.C.H. 2008 a: *Climate change and globalization in the Arctic: an integrated approach to vulnerability assessment*. Earthscan: London. pp. 254

Keskitalo, E.C.H. 2008 b: Vulnerability and adaptive capacity in forestry in northern Europe: a Swedish case study. Climatic Change. *Climatic Change*, *87*(1-2), 219-234.

Kivinen, S., Moen, J., Berg, A., & Eriksson, Å. 2010: Effects of modern forest management on winter grazing resources for reindeer in Sweden. *Ambio*, *39*(4), 269-278.

Kumpula, J., & Colpaert, A. 2009: Snow conditions and usability value of pastureland for semi-domesticated reindeer (Rangifer tarandus tarandus) in northern boreal forest area. *Rangifer*, *27*(1).

Kumpula, J., Colpaert, A., & Nieminen, M. 2000: Condition, potential recovery rate, and productivity of lichen (Cladonia spp.) ranges in the Finnish reindeer management area. *Arctic*, 152-160.

Lantto, P. 2010: Borders, citizenship and change: The case of the Sami people, 1751–2008. *Citizenship studies*, *14*(5), 543-556.

Löf, A. 2014: C*hallenging adaptability: Analysing the governance of reindeer husbandry in Sweden*. PhD thesis, Umeå.

Sandström, C., Moen, J., Widmark, C., & Danell, Ö. 2006: Progressing toward co-management through collaborative learning: forestry and reindeer husbandry in dialogue. *The International Journal of Biodiversity Science and Management*, *2*(4), 326-333.

Sandström, P., Granqvist Pahlén, T., Edenius, L., Tømmervik, H., Hagner, O., Hemberg, L.*et al.* 2003: Conflict resolution by participatory management: remote sensing and GIS as tools for communicating land-use needs for reindeer herding in northern Sweden. *Ambio*, *32*(8), 557-567.

Sara, M. N. 2009: Siida and traditional Sámi Reindeer herding knowledge. *Northern Review*, *30*, 153-178.

Sara, M. N. 2011: Land usage and siida autonomy. *Arctic Review*, *2*(2).

Skarin, A., Danell, Ö., Bergström, R., & Moen, J. 2010: Reindeer movement patterns in alpine summer ranges. *Polar biology*, *33*(9), 1263-1275.

Tveraa, T., Fauchald, P., Gilles Yoccoz, N., Anker Ims, R., Aanes, R., & Arild Høgda, K. 2007: What regulate and limit reindeer populations in Norway?. *Oikos*, *116*(4), 706-715.

Tyler, N. J. C., Turi, J. M., Sundset, M. A., Bull, K. S., Sara, M. N., Reinert, E., *et al.* 2007: Saami reindeer pastoralism under climate change: applying a generalized framework for vulnerability studies to a sub-arctic social–ecological system. *Global Environmental Change*, *17*(2), 191-206.

Ulvevadet, B. 2008: Management of reindeer husbandry in Norway–power-sharing and participation.

Widmark, C. 2009: Forestry and reindeer husbandry in northern Sweden–the development of a land use conflict. *Rangifer*, *26*(2), 43-54.

S2: Reindeer habitat selection in Norrbottens winter grazing area in dependence of forest age

To support our hypothesis of the generality of misfit, we conducted analyses on reindeer habitat selection at the landscape level in Norrbotten, Northern Sweden.
To analyse the forest variables (age and area), we extracted information about forest stand variables from the Swedish Agricultural University's database at http://skogskarta.slu.se/. These stand level data, including mean age, volume, species composition, area of the stand etc. are derived from a combination of satellite imagery (SPOT 4 and SPOT 5 taken in the year 2005, with a resolution of 25 m x 25 m). These are combined with field inventories from the Swedish National Forest Inventory (NFI). NFI reference plots are used to calculate stand variables for each pixel according to the k nearest neighbour method (kNN) (Reese *et al.* 2002). Our analysis is based on a segmented version of the kNN data set with 25 x 25 m pixels.
Land use data pertaining to reindeer husbandry are available at http://projektwebbar.lansstyrelsen.se/gis/Sv/Pages/nationella-geodata.aspx. We accessed the data files on "trivselland", i.e. areas preferred by reindeer over a longer time period, and calculated their spatial extent in a GIS.
Selective use of a resource or a habitat implies that the item is exploited disproportionately to its availability. For habitat selection at the landscape level, selection indices have been developed to describe the degree of preference and usage for a certain habitat class. These habitat classes are defined as forest age classes in our analysis (Fig. 3).
To analyze the preference for forest age classes *i*, we followed Manly *et al.* (2007). The whole winter grazing area was defined the as the *available area*, while "trivselland" was defined as *area used*.
For each forest age class *i* (< 10, 11 – 20, …, > 130) we calculated the spatial extent. Then, the *proportional availability* (A_i) of each forest age class was calculated by dividing its respective spatial extent by the whole area of analysis, i.e. the winter grazing area in Norrbotten. The availability of the forest age classes in % is also illustrated in Fig. 2a in the main text.

$$Ai = \frac{area\ of\ forest\ age\ class\ i}{area\ of\ winter\ grazing\ area}$$

Accordingly, we calculated the *proportion used* of each forest age class *i* (U_i) for stands included in the "trivselland":

$$Ui = \frac{area\ \ of\ forest\ age\ class\ i\ in\ \text{trivselland}}{area\ of\ trivselland}$$

These two proportions are used to calculate the selection index W_i for each forest class i:

$$Wi = \frac{Ui}{Ai}$$

Values of W_i greater than 1.0 represent a selective use, i.e. preference, for forest age class *i*, values less than 1.0 indicate avoidance or non-use of forest age class *i*. Results are presented in Table S2. W_i is illustrated in Fig. 2b in the main text.
The same data were used to analyze the spatial extent of forest stands in comparison to the area of "trivselland".

Table S2: Habitat selection of reindeer in Norrbottens winter grazing area: values for A_i, U_i and W_i

	Area available (A_i)		Area used (Ui)		Selection Index
Forest Age Class	Area (ha)	A_i	Area (ha)	U_i	W_i
< 10	84520	0.02	22146	0.02	1.00
11 - 20	240498	0.06	65898	0.06	1.05
21 - 30	444840	0.10	110390	0.10	0.95
31 - 40	502172	0.12	120181	0.11	0.92
41 - 50	493229	0.12	117044	0.10	0.91
51 - 60	611307	0.14	146502	0.13	0.92
61 - 70	767236	0.18	190130	0.17	0.95
71 - 80	659230	0.15	182306	0.16	1.06
81 - 90	340833	0.08	111097	0.10	1.25
91 -100	96264	0.02	35379	0.03	1.41
101 - 110	23408	0.01	10239	0.01	1.67
111 - 120	9869	0.00	4447	0.00	1.72
121 - 130	4849	0.00	2165	0.00	1.71
> 130	2030	0.00	1092	0.00	2.06
Sum	4280285			1119017	

Reference:

Manly, B. F. L., McDonald, L., Thomas, D., McDonald, T. L., & Erickson, W. P. 2007: *Resource selection by animals: statistical design and analysis for field studies*. Springer.
